# Phenolic compounds from pumpkin pulp: Extraction optimization and biological properties

**DOI:** 10.1016/j.fochx.2024.101628

**Published:** 2024-07-06

**Authors:** Nicola Pinna, Salwa Ben Abbou, Federica Ianni, Giancarlo Angeles Flores, Anne Pietercelie, Giuseppe Perretti, Francesca Blasi, Paola Angelini, Lina Cossignani

**Affiliations:** a Department of Pharmaceutical Sciences, University of Perugia, 06126 Perugia, Italy; b Department of Sciences and Techniques, Institut Meurice, 1070 Bruxelles, Belgium; c Department of Pharmacy, “Gabriele d'Annunzio” University, 66100, Chieti, Italy; d Department of Agricultural, Food and Environmental Sciences, University of Perugia, 06121 Perugia, Italy; e Department of Chemistry, Biology and Biotechnology, University of Perugia, 06100 Perugia, Italy

**Keywords:** Crop, Bioactives, Phenol extraction, HPLC-DAD analysis, Antioxidant activity, Functional foods

## Abstract

Ultrasound-assisted extraction of (poly)phenols from pumpkin pulp was optimized using response surface methodology. Time, solvent-to-sample ratio and percentage of water in ethanol were considered and a full factorial design was applied. The optimized conditions (10 min; 30 mL/g; 50% water in ethanol) were used to isolate (poly)phenols from five pumpkin varieties (Hokkaido, Lunga di Napoli, Mantovana, Moscata di Provenza, Violina rugosa), collected for two successive years. Phenolic acids were the most abundant phenolic class (gallic acid up to 413.50 μg/g), even if differences were observed among varieties and harvesting years. Generally, Violina rugosa harvested in 2021 showed the highest total phenol content (7.59 mg gallic acid equivalents/g) and antioxidant properties (16.93 mg Trolox equivalents/g). All pumpkin variety exhibited antimicrobial activity within a concentration range of 1.95 to 250 μg/mL. In conclusion, the pumpkin pulp can be considered a promising natural source of (poly)phenols for the development of health-promoting products.

## Highlights

• Isolation of (poly)phenols from pumpkin pulp was optimized by experimental design.
• Gallic and chlorogenic acids were the most abundant phenolic compounds of pulp.
• Violina rugosa showed the highest total phenol content and antioxidant properties.
• Phenolic data were useful for sample discrimination by chemometric approach.

## 1 Introduction

The consumption of vegetables and plant-based products is highly recommended for their beneficial health properties due to the presence of phytochemicals. Daily consumption of foods rich in (poly)phenols (Frank et al., 2020), carotenoids, tocopherols, sterols and other bioactives positively affects human physiological processes and metabolism (Samtiya, Aluko, Dhewa, & Moreno-Rojas, 2021).

Nowadays, researchers' interest in the in-depth study of functional foods is increasing, including pumpkins (*Cucurbita spp.*), one of the richest natural sources of nutrients and bioactive metabolites (Ahmad & Khan, 2019). Pumpkin, a vegetable from the Cucurbitaceae family, is largely used all around the world. Originally from Mexico and Guatemala, it was brought to Europe in the 16th century. All anatomical parts of pumpkin are rich in terpenoids and phenolic compounds, as well as proteins, polysaccharides, and mineral salts (Batool et al., 2022).

The main part of the pumpkin is represented by the flesh, but scientific research has so far focused mainly on the seeds, used for oil production (Hussain et al., 2021). Recently, in order to minimize agri-food loss and optimize waste management, pumpkin by-products (leaves, filaments, and peel) have been strategically exploited in various fields (Leichtweis et al., 2022; Pinna et al., 2023).

Pumpkin is employed in various sectors as a functional food, including beverages, dairy products and bakery goods. In fact, pumpkin flour may also increase the gluten network in the dough, or improve the quality and texture properties of confectionery products (Ghendov-Mosanu et al., 2023). The pulp has been described to be rich in bioactive metabolites, among which carotenoids, responsible for the significant colour changing from red to orange-yellow depending on the variety (Aziz et al., 2023; Hussain et al., 2023). Moreover, the pulp shows the intriguing presence of (poly)phenols, more polar bioactives, but lesser studied than terpenoids. The qualitative and quantitative profile of phenolic compounds present in the flesh (mainly phenolic acids, lignans, and flavones) is influenced by several factors, such as ripeness and environmental conditions (Mokhtar et al., 2021).

Based on the results of previous researches (Pinna et al., 2023; Pollini et al., 2020), ultrasound-assisted extraction (UAE) was chosen to isolate (poly)phenols from the pulp of pumpkins of five varieties (Hokkaido, Lunga di Napoli, Mantovana, Moscata di Provenza, and Violina rugosa). The aim of the research was first of all the optimization of the extraction conditions of (poly)phenols by experimental design, and then the analytical and biological characterization of the extracts. Finally, a discrimination of the samples based on harvesting year was also carried out.

## 2 Materials and methods

### 2.1 Plant materials and preparation

Pumpkins (*Cucurbita spp.*) of five different varieties, three from *C. moschata* (Lunga di Napoli, Moscata di Provenza, Violina rugosa) and two from *C. maxima* (Hokkaido, and Mantovana) species, were collected in November 2021 and 2022 in the Umbria region (Bastia Umbra, Perugia, Central Italy, altitude 180 m a.s.l., coordinates 43°04′24″N 12°33′04″E).

The peel was removed using a sharp knife, while filaments and seeds were manually separated. The pulp of pumpkins was chopped into small pieces and then dried in a ventilated oven (Binder, Series ED, Tuttlingen, Germany) at 37 °C for 24 h, and in any case until a constant weight was achieved. Finally, dried pieces were grounded in a blender and passed through a 250 μm sieve to obtain a fine powder (moisture 10 ± 1%). The samples were stored in amber glass containers protected from light and humidity at room temperature, until extraction.

### 2.2 Chemicals

Gallic acid (≥99%), chlorogenic acid (≥95%), kaempferol (≥90%), protocatechuic acid (≥99%), quercetin (≥95%), catechin (≥97%), epicatechin (≥98%), kaempferol (≥97%), ferulic acid (≥99%), *trans*-cinnamic acid (≥99%), ciprofloxacin (≥98%), fluconazole (≥98%), griseofulvin, Mueller-Hinton broth (MHB), Tryptic Soy Agar (TSA), Sabouraud Dextrose Agar (SDA), RPMI (Roswell Park Memorial Institute) 1640 medium, 2,2-diphenyl-1-picrylhydrazyl (DPPH radical), Folin and Ciocalteu's phenol reagent, and (±)-6-hydroxy-2,5,7,8-tetramethylchromane-2-carboxylic acid (Trolox) were from Sigma-Aldrich (Milan, Italy). HPLC grade and analytical grade solvents were acquired from VWR (Milan, Italy). Deionized water was obtained using a Milli-Q system (Millipore Corp, Billerica, MA, USA).

### 2.3 Ultrasound-assisted extraction (UAE) of (poly)phenols: Optimization by experimental design

The extraction of (poly)phenols from pumpkin powder was carried out at 45 °C in an ultrasonic bath (mod. AU-65; ArgoLab, Carpi, Italy), consisting of a stainless-steel jug (maximum capacity 6500 mL). Violina rugosa variety (2021) was chosen for the optimization step. The influence of some parameters on the extraction efficiency of bioactive compounds from pumpkin powder and the optimization of phenol extraction conditions were studied by experimental design, using the software MODDE 5.0™ Statistical Design Package (UMETRICS, Umeå, Sweden).

The following factors were considered:

• solvent composition: quantitative factor, expressed as H_2_O % in ethanol, with values set from 0 to 50%;
• liquid/solid ratio, indicated as L/S ratio: quantitative factor expressed as solvent volume/pulp weight in grams of dry matter (mL/g DM), with values set from 10 to 30;
• time: quantitative factor with values set from 10 to 40 min.

The following responses were selected:

• total phenolic content (TPC), expressed as milligrams of gallic acid equivalent per gram of dry matter (mg GAE/g DM);
• free radical-scavenging activity by ABTS assay, expressed as milligrams of Trolox Equivalents per gram DM (mg TE/g DM);
• ferric reducing antioxidant power (FRAP) assay, expressed as mg TE/g DM;
• gallic acid content, expressed as μg/g DM;
• chlorogenic acid content, expressed as μg/g DM.

A full factorial design (two levels) was selected, indicating a total of eleven experiments (named VRN1-VRN11, where VR indicates the Violina rugosa variety and N the experiment number) including three replicated centre points (**Table S1**). The extractions were carried out in random order according to the worksheet indicated by the software. The model was then fitted using the partial least squares regression analysis, a statistical method that bears some relation to principal components regression, finding a linear regression model by projecting the predicted variables and the observable variables to a new space. The best UAE conditions obtained for Violina rugosa were adopted for the extraction of (poly)phenols from the other varieties of pumpkin (Hokkaido, Moscata di Provenza, Mantovana, and Lunga di Napoli) (**Fig. S1**), harvested in the same farm for two successive years (2021 and 2022).

### 2.4 Determination of total phenolic content (TPC)

The TPC was determined according to the spectrophotometric method of Singleton and Rossi (1965) with some modifications (Pagano et al., 2017). The assay involved the reduction of Folin and Ciocalteu's reagent, and the absorbance was measured at 765 nm using a Lambda 20 spectrophotometer (PerkinElmer, Inc.; Waltham, Massachusetts, USA). The TPC, reported as mg GAE/g DM, was calculated from the regression equation (range of calibration 0.00625–0.1 mg/mL).

### 2.5 In vitro antioxidant activities

#### 2.5.1 DPPH assay

A DPPH assay was carried out according to the procedure reported by Pollini et al. (2019). DPPH (0.06 mM in ethanol) was added to the extract and the mixture was kept in the dark for 30 min, after which the absorbance at 517 nm was measured. The antiradical capacity against DPPH, reported as mg TE/g DM, was calculated from the regression equation (range of calibration 0.001–0.25 mg/mL).

#### 2.5.2 ABTS Assay

ABTS assay was carried out following the procedure developed by Rice-Evans and Miller (1994). Extract was added to ABTS^+•^ reagent and, after 10 min, the absorbance was measured at 734 nm. The antiradical capacity against ABTS, reported as mg TE/g DM, was calculated from the regression equation (range of calibration 0.02–0.3 mg/mL).

#### 2.5.3 FRAP Assay

The reducing capacity of the extracts was determined using the FRAP assay, according to the procedure reported by Pollini et al. (2019). The sample was added to FRAP reagent and, after 30 min, the absorbance was measured at 593 nm. The reducing capacity, expressed as mg TE/g DM, was calculated from the regression equation (range of calibration 0.0125–0.1 mg/mL).

### 2.6 HPLC-DAD analysis of phenolic compounds

The HPLC analysis of pumpkin pulp extracts was carried out according to a previous paper (Pollini et al., 2020). A pump Thermo Spectraseries, a Spectra System UV6000LP diode array detector (Thermo Separation Products, San Jose, CA, USA), and a Hypersil GOLD column (150 mm × 4.6 mm, 3 μm particle size) were used. The mobile phase consisted of 0.1% (*v*/v) formic acid in water (A) and methanol (B), with the following gradient: (B) increased from 0% to 30% in 35 min, to 40% after10 min, to 60% after 10 min, and finally, after 15 min, decreased to 30%. The flow rate was 0.7 mL/min. Xcalibur software version 1.2 (Finnigan Corporation 1998–2000, San Jose, CA, USA) was used for chromatogram and data acquisition. Peak identification was carried out with the purchased standards, confirmed through the comparison of the UV–Vis spectra with literature data, and, if necessary, through the co-injection (spiking) of selected standards in the extracts. For the quantitative HPLC-DAD analysis, calibrations curves of standard stock solutions (0.01–12.5 μg/mL) were used. Gallic acid and catechin standard solutions were injected and analyzed at 250 nm for the quantification of gallic and protocatechuic acids and of catechin and epicatechin, respectively. Chlorogenic acid standard solutions were injected and analyzed at 300 nm for the quantification of ferulic and chlorogenic acids, and at 280 nm for cinnamic acid. A calibration curve of quercetin standard was prepared setting the detector at 350 nm for the quantification of quercetin and kaempferol.

### 2.7 Antimicrobial activity

The *in vitro* antimicrobial activity of extracts from pumpkin pulp was assessed against four strains of bacteria, both Gram-negative and Gram-positive: *Escherichia coli* (ATCC 10536), *Pseudomonas aeruginosa* (ATCC 15442), *Bacillus subtilis* (PeruMyc 6), *Salmonella typhi* (PeruMyc 7). Furthermore, the extracts were also tested for their antifungal properties against various species of yeasts and dermatophytes, including *Candida tropicalis* (YEPGA 6184), *C. albicans* (YEPGA 6379), *C. parapsilopsis* (YEPGA 6551), *Trichophyton mentagrophytes* (CCF 4823), *Trichophyton tonsurans* (CCF 4834), *Arthroderma quadrifidum* (CCF 5792), *Trichophyton mentagrophytes* (CCF 5930), *Arthroderma gypseum* (CCF 6261), and *Arthroderma insingulare* (CCF 5417).

Two quality control yeast strains, *C. parapsilosis* (ATCC 22019) and *C. krusei* (ATCC 6258), were utilized in antifungal assays following guidelines outlined in D'Agostino et al. (2022). Voucher microbial cultures are preserved in the PeruMycA culture collection at the Department of Chemistry, Biology, and Biotechnology (DCBB) of the University of Perugia (Italy), and can be requested as needed. The pumpkin pulp extracts were tested for minimum inhibitory concentration (MIC) determination within the range of 1.95–250 μg/mL. Ciprofloxacin, fluconazole, and griseofulvin were included in the study in concentrations ranging from 1.56 to 200 μg/mL, 0.063–16 μg/mL, and 0.03–8 μg/mL, respectively, serving as controls for antibacterial and antifungal agents (Pagiotti et al., 2011).

In the case of pumpkin pulp extracts, the MIC end-points were determined as the lowest concentration at which no visible growth was observed (for ciprofloxacin, fluconazole, and griseofulvin, the MIC end-points were defined as the lowest concentration that inhibited 80% of the growth compared to the growth control) (Angelini et al., 2021; D'Agostino et al., 2022).

#### 2.7.1 Antibacterial susceptibility testing

The MIC of the pumpkin pulp extracts was evaluated using the broth dilution method M07-A9 established by the Clinical and Laboratory Standard Institute (CLSI, 2008). To determine the MIC, bacterial suspensions were prepared by selecting three to five colonies of the bacterial strains from 24-h cultures on TSA plates. These colonies were then pre-grown overnight in MHB to achieve a cell density of approximately 1–2 × 10^8^ colony-forming units per milliliter (CFU/mL), equivalent to the 0.5 McFarland standard.

The bacterial suspensions were diluted in fresh MHB and added to the MIC dilution series to reach a concentration of 5 × 10^5^ CFU/mL in each tube in accordance with CLSI M07-A9 guidelines. This was confirmed by plating serial dilutions of the inoculum suspensions on MHA. Controls included MHB-grown bacterial cultures for viability, as well as uninoculated MHB with pumpkin pulp extracts for incubation. MIC endpoints were determined after 18–20 h of incubation at 35 °C in ambient air, as described by Pagiotti et al. (2011). Geometric means and MIC ranges were determined from the three biological replicates to allow comparisons between the activities of pumpkin pulp extracts.

#### 2.7.2 Antifungal susceptibility testing

Susceptibility testing for yeasts and filamentous fungi was carried out following the CLSI M27-A3 and M38-A2 protocols (D'Agostino et al., 2022). The study used RPMI 1640 medium, which was sodium bicarbonate-free and enriched with 2% glucose (*w*/*v*), l-glutamine, and buffered with 0.165 mol/L 4-morpholinepropanesulfonic acid at pH 7.0.

In summary, fungal inocula were prepared from 7-day-old cultures on SDA at 25 °C and adjusted to optical densities (OD 600) between 0.09 and 0.11 (MacFarland standard). Inoculum sizes ranging from 0.2 to 0.4 × 10^4–5^ CFU/mL were achieved by diluting filamentous fungi and yeast inoculum suspensions to a 1:50 ratio in RPMI 1640. The accuracy of the inoculum sizes was confirmed by plating serial dilutions on SDA. MIC endpoints (μg dry extracts/mL) were determined after 24 h (for yeasts) and 72 h (for dermatophytes) of incubation at 30 °C in ambient air (D'Agostino et al., 2022). Geometric means and MIC ranges were calculated from three biological replicates to allow for comparison of the effectiveness of pumpkin pulp extracts.

### 2.8 Data analysis

All the analytical procedures were carried out in triplicate, and the results were expressed as mean ± standard deviation (SD) on dry weight (DW). Statistical significance (*p* < 0.01) among eleven samples (VNR1-VNR11) and among the five varieties was measured using one-way analysis of variance (ANOVA) followed by Tukey's honestly significant difference *post hoc*. Statistical significances (*p* < 0.01) between the two harvesting years of each variety, as well as between the two years of all varieties, were measured using Student's *t*-test. Principal component analysis (PCA) and linear discriminant analysis (LDA) were used for the differentiation and classification of samples. Data were processed and edited with Microsoft Excel 2016 (Microsoft Office, WA, USA) and XLSTAT (Lumivero, 2024) software.

## 3 Results and discussion

### 3.1 Optimization of extraction conditions of (poly)phenols from pumpkin pulp powder by UAE

In this research, an optimization study for the extraction of phenolic compounds from pumpkin was carried out by experimental design, a technique for planning experiments, used to minimize their number when several experimental parameters simultaneously change. Based on the experimental data, a mathematical model of the studied process is generated. It is useful to understand the influence of experimental parameters and their interactions on selected responses and to find the optimal conditions for the investigated process, in this case the extraction of (poly)phenols from pumpkins of different variety and harvesting year.

To evaluate the influence of UAE conditions on TPC, antioxidant activity (ABTS, FRAP, and DPPH), and two main phenolic compounds (gallic and chlorogenic acids), the software MODDE 5.0™ was used. In this work, pure ethanol and hydroalcoholic solutions were used as extraction solvents. The ratio solvent/sample (mL/g DM), indicated as the liquid/solid ratio (L/S; 30:1, 20:1, and 10:1), and the time (ranging from 10 to 40 min) were also considered because important parameters that influence extraction.

The quality of the mathematical model, obtained with spectrophotometric and chromatographic results, was evaluated by two statistical parameters, R^2^ (which describes how well the model fits the experimental data) and Q^2^ (which describes how well the model will predict new data), showed in **Table S2**. The developed model can be considered good and therefore useful for optimization and prediction since the R^2^ values ranged from 0.918 for DPPH to 0.998 for FRAP, while the Q^2^ values ranged from 0.649 for DPPH to 0.830 for TPC.

Initially, the extraction yield was determined *via* gravimetric method and the results showed the highest values for VRN4 and VRN8 samples (67.48 and 67.87%, respectively), both obtained using a water % of 50 and a liquid/solid ratio of 30 mL/g. Instead, the lowest values of yield (10.17 and 14.84%) were found for extracts (VRN1 and VNR5, respectively) obtained without water and with a L/S ratio of 10 mL/g.

Table 1 shows the values of the responses of the eleven experimental samples, obtained with the extraction conditions reported in **Table S1**. It can be observed that samples VRN4 and VRN8 showed the highest values of TPC and antioxidant parameters, while samples VNR1 and VNR5 the lowest. In this research three extraction parameters (% water in ethanol, L/S ratio, time) were investigated and the **Fig. S2 (a-d)** shows the coefficients of the selected factors for TPC (A), DPPH (B), ABTS (C), and FRAP (D) responses, indicating the influence of the extraction factors on the considered responses. Moreover, **Table S3** shows the coefficients of the extraction factors on the content of gallic and chlorogenic acids. It can be observed that the factors % water in ethanol and L/S ratio positively influenced the response, with a greater effect for water content compared to the ratio. These results indicated that pumpkin extracts with the highest TPC value and antioxidant properties can be obtained with the highest percentage of water (50%) in the hydroalcoholic mixture and with the highest L/S ratio (30:1). It should be emphasized that time showed negligible influence on the response, and this means that a rapid and cost-effective extraction can be done without affecting the extraction yield. Therefore, ten minutes can be sufficient for a good extraction of (poly)phenols from pulp powder.

**Table 1:** Experimental values of TPC, ABTS, FRAP, gallic and chlorogenic acids of the eleven UAE extracts (VRN1-VRN11).

	**TPC**	**DPPH**	**ABTS**	**FRAP**	**Gallic acid**	**Chlorogenic acid**
	mg GAE/g	mg TE/g	mg TE/g	mg TE/g	μg/g	μg/g
VRN1	1.06 ± 0.07^a^	0.34 ± 0.00^a^	1.76 ± 0.20^a^	0.50 ± 0.01^a^	79.94 ± 12.71^a^	62.28 ± 8.03^a^
VRN2	6.01 ± 0.15^b^	1.38 ± 0.12^b^	18.00 ± 0.19^b^	2.81 ± 0.03^b^	357.97 ± 10.81^b^	192.77 ± 3.78^b^
VRN3	1.41 ± 0.00^c^	0.33 ± 0.02^a^	4.20 ± 0.35^c^	0.75 ± 0.03^a,d^	62.67 ± 12.65^c^	78.49 ± 10.19^c^
VRN4	7.48 ± 0.03^d^	1.94 ± 0.15^c^	19.56 ± 0.49^b^	3.87 ± 0.19^c^	428.87 ± 5.16^d^	251.31 ± 2.20^d^
VRN5	1.39 ± 0.03^c^	0.59 ± 0.03^a^	4.37 ± 0.15^c^	0.78 ± 0.02^d^	78.73 ± 0.37^e^	106.21 ± 2.68^e^
VRN6	5.94 ± 0.06^b,f^	1.27 ± 0.11^b^	14.95 ± 0.62^d,f^	3.35 ± 0.25^e,f^	182.18 ± 38.26^f^	176.44 ± 5.01^f^
VRN7	2.16 ± 0.03^e^	0.41 ± 0.04^a^	5.15 ± 0.03^c^	0.96 ± 0.02^d^	101.30 ± 3.08^g^	113.26 ± 28.90^e,g^
VRN8	7.65 ± 0.18^d^	1.32 ± 0.01^b^	22.60 ± 1.32^e^	4.11 ± 0.08^c^	240.86 ± 4.11^h^	182.48 ± 2.15^f,h^
VRN9	5.82 ± 0.18^f^	1.93 ± 0.25^c^	14.92 ± 0.96^f^	3.23 ± 0.09^f^	306.26 ± 1.77^i^	183.75 ± 7.03^f,h^
VRN10	6.62 ± 0.42^g^	1.90 ± 0.04^c^	18.21 ± 0.45^b^	3.56 ± 0.11^e^	296.76 ± 31.51^i,l^	189.36 ± 11.42^f,h^
VRN11	6.77 ± 0.16^g^	1.93 ± 0.09^c^	18.18 ± 1.65^b^	3.57 ± 0.11^e^	364.53 ± 5.09^b,m^	214.86 ± 14.73^i^

Data are reported as mean value ± standard deviation of three independent measurements (*n* = 3) and are expressed on dry weight (DW). Values with different lowercase letters in the same column indicate significant differences (*p* < 0.01).

TPC, total phenol content; DPPH, 2,2-diphenyl-1-picrylhydrazyl; ABTS; 2,2′-azino-bis(3-ethylbenzothiazoline-6-sulphonic acid) diammonium salt; FRAP, ferric reducing antioxidant power; GAE, gallic acid equivalent; TE, Trolox equivalent; VRN, Violina rugosa number (where VR indicates the Violina rugosa variety and N the number of the experiment)

**Fig. 1:**
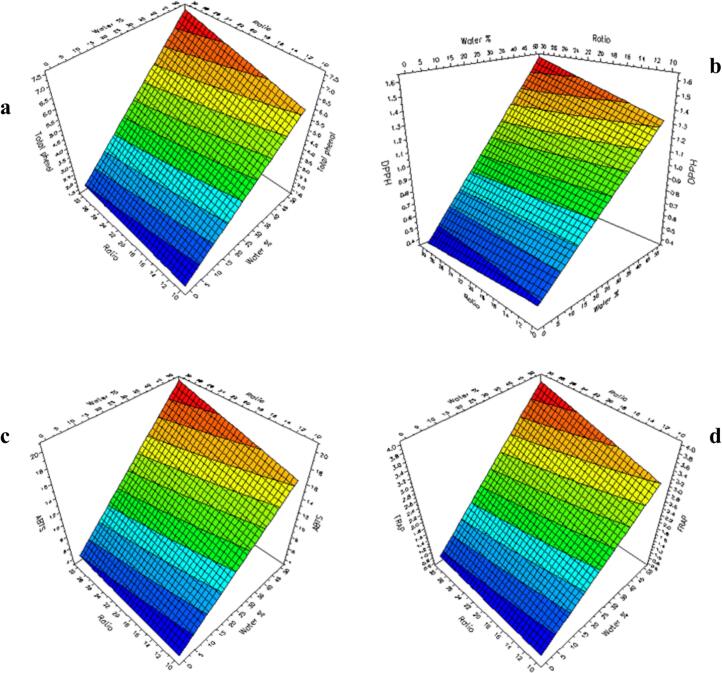
Ultrasound-assisted extraction experimental design. Surface plot showing the responses: a) TPC; b) DPPH; c) ABTS; d) FRAP.

Fig. 1**(a-d)** shows the surface plots generated by the software with the four responses, TPC (a), DPPH (b), ABTS (c) FRAP (d), as a function of two selected factors for UAE experiments (water % and L/S ratio), setting the time value constant. The response-surface plot is generated to obtain a graphical representation of the experimental region, showing that the change of colour from blue to red means an increase in the response. Based on this consideration, it can be stated that the increase in the % of water in ethanol (from 0 to 50%) and the L/S ratio (from 10 to 30 mg/L), corresponded to an increase in the TPC value, as well as in the antioxidant properties determined by the three complementary spectrophotometric assays. A correlation study was also carried out, considering the results of all spectrophotometric parameters and TPC-HPLC of the VRN1-VRN11 extracts (**Table S4**). Interestingly, good correlations were always obtained (R^2^ ≥ 0.7652), and the best correlation values were found for TPC *vs* FRAP (R^2^ = 0.9878) and TPC *vs* ABTS (R^2^ = 0.9808). Good values of correlation (R^2^ ≥ 0.7853) were always obtained between TPC-HPLC and spectrophotometric data.

### 3.2 Total phenol content and antioxidant properties of different varieties of pumpkin

The next part of the study concerned the extraction, by optimized UAE procedure, of (poly)phenols from the pulp powder of different varieties of pumpkin (Hokkaido, Lunga di Napoli, Mantovana, Moscata di Provenza, and Violina rugosa), purchased in 2021 and 2022 in the same growing area. Based on the results of the optimization step, the following extraction conditions were used: 50% water in ethanol, L/S ratio 30:1, time 10 min. Regarding the extraction yield, similar results were obtained for pumpkins purchased in different years (57.69–66.43% for 2021; 52.58–68.01% for 2022), with Mantovana (2022) which gave the highest extraction yield (68.01%) and Hokkaido (2022) the lowest (52.58%).

Table 2 shows the TPC and antioxidant properties of the extracts. As regards spectrophotometric assays, it can be observed that Violina rugosa provided the highest values of TPC for both years, whereas Lunga di Napoli exhibited the lowest amounts of TPC for 2021 and Moscata di Provenza for 2022. Additionally, similar TPC values for Moscata di Provenza in both years were obtained, while the other varieties showed different amounts of phenolic content in each year. In regards to antioxidant properties, Lunga di Napoli (2021) exhibited the lowest values of DPPH, ABTS, and FRAP for 2021 year, while Violina rugosa (2022) the highest for 2022 year. However, a noticeable variation in TPC value and antioxidant activity was observed based on the year of pumpkin harvesting. Overall, phenolic content was higher in the 2021 samples than in the 2022 samples, and all pumpkins purchased in 2021 demonstrated higher levels of antioxidant activity than those purchased in 2022. Since environmental factors including temperature and rainfall might affect the synthesis of bioactive components by plants, some data on weather conditions were detailed. The temperature in the period April–August was higher in 2022 in respect to 2021. Furthermore, the total rainfall during the two harvesting years was similar (587.0 *vs* 591.0 mm for 2022 and 2021, respectively), however some differences were recorded in the months of July and August with low rainfall in 2021 compared to 2022 (9.6 *vs* 35 mm) (Regione Umbria, 2021, 2022).

**Table 2:** Value of TPC and antioxidant assays (DPPH, ABTS, FRAP) of extracts from five pumpkin varieties, collected for two successive years (2021−2022).

	**Year**	**TPC**	**DPPH**	**ABTS**	**FRAP**
		mg GAE/g	mg TE/g	mg TE/g	mg TE/g
Hokkaido	2021	6.80 ± 0.27^a^	3.50 ± 0.24^a^	9.54 ± 0.03^a^	4.05 ± 0.04^a^
	2022	3.99 ± 0.02^b^	0.26 ± 0.01^b^	7.90 ± 0.45^b^	1.20 ± 0.01^b^
Lunga di Napoli	2021	1.49 ± 0.10^a^	0.07 ± 0.01^a^	1.64 ± 0.09^a^	0.48 ± 0.01^a^
	2022	3.11 ± 0.05^b^	0.72 ± 0.06^b^	8.47 ± 0.15^b^	1.19 ± 0.01^b^
Mantovana	2021	2.13 ± 0.07^a^	1.14 ± 0.13^a^	3.92 ± 0.11^a^	1.56 ± 0.01^a^
	2022	3.78 ± 0.11^b^	0.68 ± 0.00^b^	9.20 ± 0.09^b^	1.17 ± 0.03^b^
Moscata di Provenza	2021	2.91 ± 0.05^a^	1.05 ± 0.00^a^	4.98 ± 0.21^a^	1.79 ± 0.00^a^
	2022	2.68 ± 0.07^b^	0.43 ± 0.02^b^	7.87 ± 0.57^b^	0.81 ± 0.01^b^
Violina rugosa	2021	7.59 ± 0.22^a^	2.93 ± 0.15^a^	16.93 ± 1.42^a^	4.70 ± 0.09^a^
	2022	4.98 ± 0.07^b^	0.78 ± 0.0^b^	10.52 ± 0.83^b^	1.40 ± 0.03^b^

Data are reported as mean value ± standard deviation of three independent measurements (n = 3) and are expressed on dry weight (DW). Values with different lowercase letters in the same column (same variety, different years) indicate significant differences (*p* < 0.01). TPC, total phenol content; DPPH, 2,2-diphenyl-1-picrylhydrazyl; ABTS; 2,2′-azino-bis(3-ethylbenzothiazoline-6-sulphonic acid) diammonium salt; FRAP, ferric reducing antioxidant power; GAE, gallic acid equivalent; TE, Trolox equivalent

For example, the 2021 samples showed a greater capacity to scavenge of the DPPH radical than those of the following year, in fact the highest value of DPPH in 2021 was 3.50 mg TE/g (Hokkaido), while in 2022 was 0.78 mg TE/g (Violina rugosa). The ABTS values in 2021 ranged from 1.64 mg TE/g of Lunga di Napoli to 16.93 mg TE/g of Violina rugosa, while in 2022 ranged from 7.87 mg TE/g of Moscata di Provenza to 10.52 mg TE/g of Violina rugosa. In line with ABTS trend, the FRAP values in 2021 were the lowest for Lunga di Napoli (0.48 mg TE/g) and the highest for Violina rugosa (4.70 mg TE/g), and in 2022 Moscata di Provenza showed the lowest values (0.81 mg TE/g) and Violina rugosa the highest (1.40 mg TE/g). It can be affirmed that Violina rugosa always showed the highest TPC, ABTS and FRAP values for both years, while, for this variety, DPPH was the highest only in 2022. Moreover, statistical analysis revealed that both year and variety significantly influenced the phenolic content and antioxidant activity, resulting in variability among the studied pumpkins. Statistically different results (*p* < 0.01) were obtained by comparing the TPC, DPPH, ABTS, and FRAP values for each pumpkin variety in the following two years. As regards the comparison among all varieties harvested in the same year, the values of TPC, DPPH, ABTS, and FRAP were always different (*p* < 0.01) with some exceptions. Regarding the varieties harvested in 2022, Mantovana and Hokkaido showed no significant differences in TPC (*p*-value = 0.038) and FRAP (*p*-value = 0.1125). Considering the varieties harvested in 2021, DPPH values of Mantovana and Moscata di Provenza were not different (*p*-value = 0.1988), as were Hokkaido and Violina rugosa (*p*-value = 0.0115). The FRAP values of Lunga di Napoli 2022 showed no significant differences with Hokkaido (*p*-value = 0.1440) and Mantovana (*p*-value = 0.2666).

The results of Kulczyński, Gramza-Michałowska, and Królczyk (2020) showed that the highest value of TPC was found for Melonowa Žółta variety for hydroalcoholic (methanol:water, 80:20, *v/v*) and aqueous extract (232.5 and 255.69 mg GAE/100 g DW, respectively). In line with this trend, DPPH, ABTS and FRAP also showed the highest value for this variety. Recently, the research group of Stryjecka, Krochmal-Marczak, Cebulak, and Kiełtyka-Dadasiewicz (2023) obtained a TPC value ranging from 62.91 to 477.89 mg GAE/100 g fresh weight (FW). The lowest values of TPC, DPPH, and FRAP were found for Kamo Kamo (*C. pepo*), while the highest values were reported for Bambino variety, belonging to *C. maxima* species. Kostecka-Gugała, Kruczek, Ledwożyw-Smoleń, and Kaszycki (2020) studied eighteen *Cucurbita* cultivars and found the highest values of FRAP and DPPH for Hokkaido variety.

### 3.3 Chromatographic characterization of different varieties of pumpkin

**Fig. 2:**
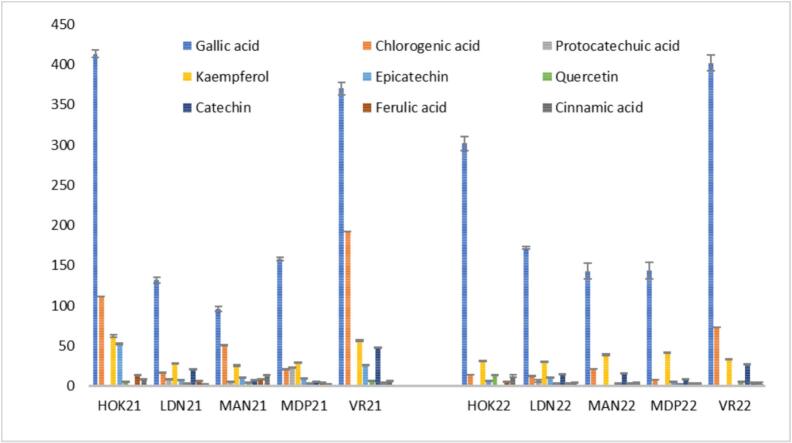
Content of (poly)phenols (μg/g) of pulp of five pumpkin varieties harvested in 2021 and 2022.

An HPLC-DAD characterization of the phenolic extracts from the five pumpkin varieties harvested in two years was also carried out. Fig. 2 shows the main phenolic compounds identified and quantified in the extracts. **Table S5** shows the retention time and spectroscopic data (UV–vis, λ_max_) used for the identification of individual compounds. **Fig. S3** shows the HPLC-DAD chromatograms extracted at 250, 300 (280 nm for cinnamic acid) and 350 nm, while Fig. **S4** shows on-line DAD-UV–Vis spectra of the identified compounds. Specifically, some compounds (gallic, cinnamic, chlorogenic and ferulic acids, kaempferol, and quercetin) were detected in all pumpkin varieties, while other phenolic compounds (epicatechin, catechin, and protocatechuic acid) only in some samples. Generally, all pumpkin varieties showed a higher content of phenolic acids (*i.e.,* gallic acid and protocatechuic acids; 68.8–86.3%) compared to flavonoids (12.2–27.0%) and hydroxycinnamic acids (1.3–9.8%) (**Figs. S5-S6** for pumpkins harvested in 2021 and 2022, respectively).

The qualitative and quantitative HPLC-DAD analysis of (poly)phenols confirmed the variation in their profiles and contents, between different varieties and years of harvesting. The variation of the phenolic profile could be attributed to different factors, including distinct environmental conditions that affect the synthesis of bioactive components by the plants (Kostecka-Gugała et al., 2020). Moreover, work performed on vegetable foods or byproducts, based on UAE extraction, has shown that factors such as solvent composition, extraction time and temperature, and technical characteristics of the ultrasound equipment (power, frequency) had a great impact on the efficiency of extraction, and therefore on the chemical composition and bioactivity of the extracts (Alara, Abdurahman, & Ukaegbu, 2021; Kainat et al., 2022; Kulczyński et al., 2020; Pollini et al., 2020).

Interestingly, a correlation study was also carried out, considering all extracts and all spectrophotometric parameters as well as TPC determined by HPLC (TPC-HPLC) (**Table S6**). Good correlations were obtained (R^2^ ≥ 0.6906) when TPC values were correlated with antioxidant properties and TPC-HPLC, and the best correlation value was found for DPPH *vs* FRAP (R^2^ = 0.9307).

As regards phenol profiling, phenolic acids (gallic, protocatechuic, ferulic), chlorogenic acid, as well as flavonols (kaempferol and quercetin), were found in the samples analyzed in this research. Other authors also reported the cited compounds (Dragovic-Uzelac, Delonga, Levaj, Djakovic, & Pospisil, 2005; Krstić et al., 2023; Kulczyński & Gramza-Michałowska, 2019; Mokhtar et al., 2021), showing a wide range of variability in the content due to the different cultivars investigated. Among the recent works available on this topic, Stryjecka et al. (2023) must be cited. They reported that the phenolic acid class was the most abundant, with syringic acid as the most representative (0.44–6.61 mg/100 g FW). The same findings were reported by Kostecka-Gugała et al. (2020). They quantified eight phenolic acids and found that syringic acid was the most abundant (up to16.41 mg/100 g FW), followed by salicylic acid (up to 2.74 mg/100 g FW). Among flavonols, significant amounts of catechin were reported by Kostecka-Gugała et al. (2020) for some varieties of *C. maxima* species.

The influence of ripeness on the phenolic content and composition, as well as on antioxidant and antimicrobial activities of pumpkins (*C. moschata* Duchesne) was studied by Mokhtar et al. (2021). They found that mature fruits showed higher TPC and ABTS values than young fruits and the presence of catechin.

Finally, it must be highlighted that the variety of fruits/vegetables and the year of harvesting significantly affect the phenolic content and composition. This phenomenon is closely linked to the specific growth conditions of the vegetables (Sharma et al., 2019). From a nutritional point of view, it should be underlined that, although deficiencies in phenol intake do not lead to specific diseases, an adequate daily intake of phenolic components could benefit human health, especially in preventing chronic diseases (Fraga, Croft, Kennedye, & Tomás-Barberán, 2019).

### 3.4 Discriminant analysis

Chemometrics is the discipline that uses mathematical and statistical methods to design or select optimal measurement procedures and experiments, in fact chemometric methods allow handling complex chemical data, and maximizing the extraction of the available and useful information they encode (Biancolillo, D'Archivio, Marini, & Vitale, 2022).

In this paper, in order to make a comparison between pumpkin varieties harvested in two different years, two multivariate statistic approaches were applied (principal component analysis and linear discriminant analysis, PCA and LDA, respectively). The chemometric model (dataset) was built by using spectrophotometric (TPC, ABTS, FRAP, and DPPH) data reported in Table 2, and the contents of gallic and chlorogenic acids determined by chromatographic method, reported in Fig. 2.

**Fig. 3:**
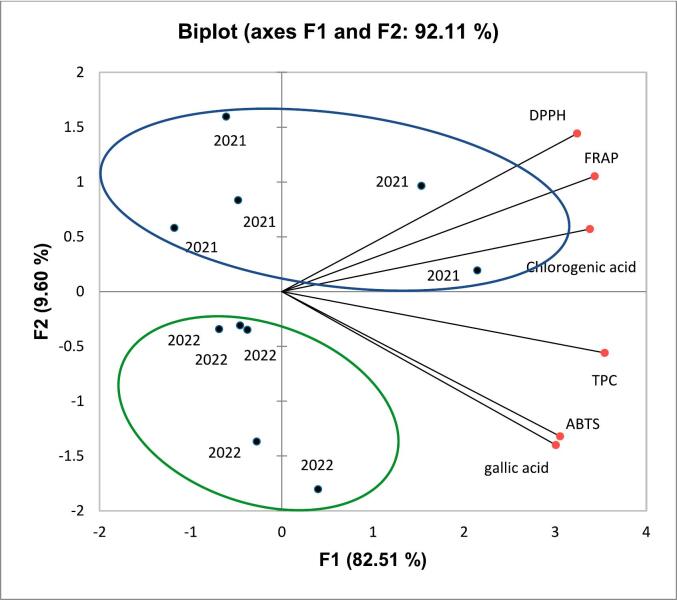
Biplot for principal components function 1 (F1) and function 2 (F2). In blue the samples harvested in 2021, in green the samples harvested in 2022. (For interpretation of the references to colour in this figure legend, the reader is referred to the web version of this article.)

PCA is a dimensionality reduction and machine learning method used to simplify a large data set into a smaller set while maintaining significant patterns and trends. **Table S7** shows eigenvalue, percentage of variance, and cumulative percentage of the principal components, while Fig. 3 shows the biplot of the PCA with vectors of each variable and the distribution of samples in the plane defined by the values of the two principal components. The first two components explained 92.11% of the total variance, showing a different location in the plane of pumpkin varieties harvested in 2021 in respect to pumpkin varieties harvested in 2022. The responsible variables were TPC, DPPH, ABTS, FRAP, chlorogenic acid, and gallic acid.

**Fig. 4:**
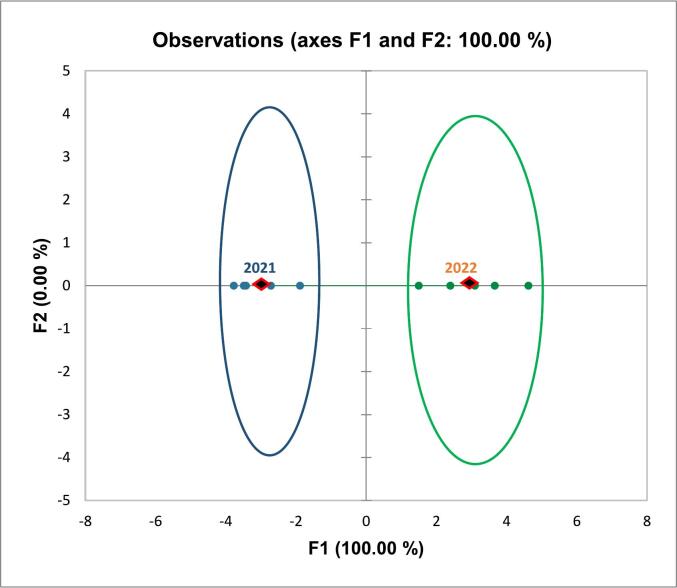
Pumpkin samples on discriminant function F1 score plot. In blue the samples harvested in 2021, in green the samples harvested in 2022. (For interpretation of the references to colour in this figure legend, the reader is referred to the web version of this article.)

LDA is an approach used in supervised machine learning to solve multi-class classification problems, separating multiple classes with multiple features through data dimensionality reduction. LDA works by identifying a linear combination of features that separates or characterizes two or more classes of objects or events. Fig. 4 shows the plot of the observations on the discriminant function axis, using spectrophotometric and chromatographic data, allowing to confirm that the two harvesting years were very well discriminated on the F1 axis. The centroid coordinates on F1 axis were 5.785 and - 5.785 for the two years, respectively. **Table S8** summarizes the prior and posterior classification, membership probabilities, scores and squared distances, showing that each observation is classified into the group for which the membership probability is greatest. The results of classification (training samples and cross-validation results), reported in **Table S9**, showed that 100% of original grouped cases were correctly classified for training samples. On the contrary, from the cross-validation results, it can be observed that the 90.0% of cross-validated group cases were correctly classified because a sample harvested in 2021 (*i.e.*, Violina rugosa) was classified as harvested in 2022. The obtained results confirm that the phenolic compounds in pumpkin, and their properties, are strongly influenced by the harvesting year.

### 3.5 Antimicrobial activity

Table 3, Table 4 present the MIC values of extracts from various pumpkin varieties against bacteria, yeasts, and dermatophytes, respectively. All pumpkin variety extracts exhibited antimicrobial activity within a concentration range of 1.95 to 250 μg/mL. Particularly, *Escherichia coli* (ATCC 10536), *Bacillus subtilis* (PeruMycA 6), and *Salmonella typhi* (PeruMycA 7) displayed the highest sensitivity to the Mantovana 2021 extract, with an MIC range of 31.25–62.5 μg/mL (GM, 39.37 μg/mL). Conversely, *Salmonella typhi* (ATCC 15442) and *Staphylococcus aureus* (ATCC 6538) exhibited limited sensitivity to nearly all sample extracts (Table 3). Generally, Gram-negative bacterial strains, including *E. coli* (ATCC 10536), *P. aeruginosa* (ATCC 15442), and *S. typhi* (PeruMycA7), displayed lower sensitivity to plant extracts compared to Gram-positive strains. The observed variations in antibacterial activity among pumpkin variety extracts against Gram-negative and Gram-positive bacteria may be attributed to differences in their cell surfaces, as previously noted by Tamboli and Lee (2013). In fact, Gram-negative bacteria possess an additional layer that plays a vital role in protecting them from hostile environments by excluding toxic molecules without compromising the exchange of materials necessary for sustaining life (Delcour, 2009). Similar findings were reported for *Fuscoporia torulosa* and *Pleurotus spp.* mushrooms (Covino et al., 2019; Angeles Flores et al., 2022; Angeles Flores et al., 2023).

**Table 3:** Minimal inhibitory concentrations (MIC) of *Cucurbita spp.* extracts against bacteria isolates.

**Variety**	**Year**	**Bacteria - MIC (**μg/mL)*					
		*Escherichia*	*Bacillus*	*Pseudomonas*	*Bacillus*	*Salmonella*	*Staphylococcus*
		*coli*	*cereus*	*aeruginosa*	*subtilis*	*typhi*	*aureus*
		(ATCC 10536)	(PeruMycA 4)	(ATCC 15442)	(PeruMycA 6)	(PeruMycA 7)	(ATCC 6538)
Hokkaido	2021	198.42(125–250)	49.60(31.25–62.5)	198.42(125–250)	157.49(125–250)	198.42(125–250)	>250
	2022	198.42(125–250)	78.74(62.5–125)	198.42(125–250)	157.49(125–250)	198.42(125–250)	>250
Lunga di Napoli	2021	78.74(62.5–125)	49.60(31.25–62.5)	157.49(125–250)	198.42(125–250)	157.49(125–250)	>250
	2022	157.49(125–250)	198.42(125–250)	198.42(125–250)	198.42(125–250)	250- > 250	>250
Mantovana	2021	39.37(31.25–62.5)	99.21(62.5–125)	99.21(62.5–125)	39.37(31.25–62.5)	49.60(31.25–62.5)	>250
	2022	198.42(125–250)	49.60(31.25–62.5)	99.21(62.5–125)	78.74(62.5–125)	198.42(125–250)	>250
Moscata di Provenza	2021	157.49(125–250)	78.74(62.5–125)	198.42(125–250)	198.42(125–250)	157.49(125–250)	250- > 250
	2022	49.60(31.25–62.5)	49.60(31.25–62.5)	250- > 250	78.74(62.5–125)	250- > 250	>250
Violina Rugosa	2021	78.74(62.5–125)	99.21(62.5–125)	39.37(31.25–62.5)	99.21(62.5–125)	250- > 250	>250
	2022	78.74(62.5–125)	99.21(62.5–125)	198.42(125–250)	157.49(125–250)	198.42(125–250)	>250
Ciprofloxacin (μg/mL)		31.49 (25–50)	125.99 (100−200)	125.99 (100–200)	125.99 (100–200)	79.37 (50–100)	200- > 200

* MIC values are reported as geometric means of three independent replicates (n = 3).

MIC range concentrations are reported within brackets.

**Table 4:** Minimal inhibitory concentrations (MIC) of *Cucurbita spp.* extracts against yeast and dermatophyte isolates.

**Variety**	**Year**	**Yeast - MIC (**μg/mL)*			
		*Candida*	*Candida*	*Candida*	*Candida*
		*tropicalis*	*albicans*	*parapsilosis*	*albicans*
		(YEPGA 6184)	(YEPGA 6379)	(YEPGA 6551)	(YEPGA 6183)
Hokkaido	2021	>250	>250	250- > 250	>250
	2022	>250	>250	>250	250- > 250
Lunga di Napoli	2021	>250	>250	250- > 250	250- > 250
	2022	>250	>250	250- > 250	198.42 (125–250)
Mantovana	2021	>250	>250	250- > 250	200- > 250
	2022	>250	>250	>250	250- > 250
Moscata di Provenza	2021	>250	>250	250- > 250	>250
	2022	>250	>250	>250	250- > 250
Violina Rugosa	2021	>250	>250	>250	250- > 250
	2022	>250	>250	250- > 250	>250
Fluconazole (μg/mL)		2	1	4	2

		Dermatophyte - MIC (μg/mL)*			
		*Trichophyton*	*Trichophyton*	*Arthroderma*	*Trichophyton*
		*mentagrophytes*	*tonsurans*	*quadrifidum*	*mentagrophytes*
		(CCF 4823)	(CCF 4834)	(CCF 5792)	(CCF 5930)
Hokkaido	2021	250- > 250	250- > 250	>250	>250
	2022	>250	250- > 250	>250	>250
Lunga di Napoli	2021	250- > 250	250- > 250	>250	>250
	2022	>250	250- > 250	>250	>250
Mantovana	2021	157.49 (125–250)	250- > 250	250- > 250	250- > 250
	2022	157.49 (125–250)	157.49 (125–250)	250- > 250	>250
Moscata di Provenza	2021	49.60 (31.25–62.5)	198.42 (125–250)	250- > 250	>250
	2022	78.74 (62.5–125)	250- > 250	>250	>250
Violina rugosa	2021	157.49 (125–250)	198.42 (125–250)	>250	250- > 250
	2022	78.74 (62.5–125)	250- > 250	157.49 (125–250)	>250
Griseofulvin (μg/mL)		2.52 (2–4)	0.198 (0.125–0.25)	>8	3.174 (2–4)

* MIC values are reported as geometric means of three independent replicates (n = 3).

MIC range concentrations are reported within brackets

Remarkably, only extracts from Lunga di Napoli 2022 pumpkins displayed significant inhibition (MIC 124–250 μg/mL) against *Candida albicans* (YEPGA 6183) (Table 4). Moreover, all tested extracts hindered dermatophyte growth, with *Trichophyton mantagrophytes* (CCF 4823) and *T. tonsurans* (CCF 4834) being the most susceptible fungal species to pumpkin extracts, with MIC values ranging from 31.25 to 250 μg/mL (Table 4). The MIC values of ciprofloxacin, fluconazole, and griseofulvin for strains *C. parapsilosis* (ATCC 22019) and *C. krusei* (ATCC 6258) fell within established ranges (D'Agostino et al., 2022).

MIC values below 100 μg/mL typically indicate potent antimicrobial activity (Webster, Taschereau, Belland, Sand, & Rennie, 2008). In this study, MIC values were occasionally lower than 100 μg/mL, suggesting a moderate antimicrobial activity of the investigated extracts. In contrast, Krstić et al. (2023) emphasized that during the assessment of antimicrobial activity *via* broth microdilution testing in 96-well microtiter plates, only *C. maxima* 117 among four pumpkin extracts exhibited moderate suppression of *S. aureus* and *S. epidermidis* growth, with an MIC of 1000 μg/mL. However, comparing bioactivity results across different studies presents challenges owing to the diverse array of extraction methods, test organisms, and testing systems employed (Dresch et al., 2015).

The antimicrobial activity of plant extracts has been demonstrated mainly *in vitro*. Nevertheless, some aspects are crucial for its use in foods. Gallic acid, caffeic acid, and ferulic acid had better antimicrobial activity against Gram-positive and Gram-negative bacteria than gentamicin and streptomycin. Some (poly)phenols, such as those found in green and black tea, can inhibit the growth of detrimental bacteria such as *Helicobacter pylori*, *S. aureus*, *Escherichia coli*, *Salmonella typhimurium*, *Listeria monocytogenes*, and *Pseudomonas aeruginosa*, as well as hepatitis C virus, influenza, HIV, and *Candida* (Lobiuc et al., 2023).

## 4 Conclusion

In this research, an experimental design approach was successfully applied for investigating the effect of some experimental variables on UAE extraction of (poly)phenols from *Cucurbita spp.* pulp. UAE optimized technique has proven to be a simple and efficient method for the extraction of thermolabile compounds, such as phenol compounds. Different varieties of pumpkin, purchased in two successive years, showed significant differences regarding phenol profile and bioactivity. Generally, it can be stated that pumpkin pulp has high concentrations of hydroxybenzoic acids, secondary metabolites produced by plants to adapt to new environments and external stress factors. The same pumpkin variety showed different phenolic content, profile and antioxidant activity in different years, supporting the fact that environmental conditions play key roles in biosynthetic pathways and demonstrating that phenolic content can be indicative of the level of stress endured by the vegetables during their growth. It can be concluded that pumpkin pulp powder can be considered a promising natural source of bioactive compounds with antioxidant activity, which can be exploited for the development of health-promoting products in nutraceutical, cosmetic and food field, even if the effect of *in vitro* digestion should be taken into consideration to better evaluate *in vivo* bioactivity.

## CRediT authorship contribution statement

**Nicola Pinna:** Formal analysis, Data curation. **Salwa Ben Abbou:** Formal analysis, Data curation. **Federica Ianni:** Validation, Investigation, Formal analysis. **Giancarlo Angeles Flores:** Formal analysis, Data curation. **Anne Pietercelie:** Methodology, Conceptualization. **Giuseppe Italo Francesco Perretti:** Methodology, Conceptualization. **Francesca Blasi:** Writing – review & editing, Writing – original draft, Methodology, Conceptualization. **Paola Angelini:** Writing – original draft, Investigation, Formal analysis. **Lina Cossignani:** Writing – review & editing, Writing – original draft, Supervision, Funding acquisition, Conceptualization.

## Declaration of competing interest

The authors declare that they have no known competing financial interests or personal relationships that could have appeared to influence the work reported in this paper.

## Data Availability

Data will be made available on request.

## Appendix A Supplementary data

Supplementary material Image 1
