# Supplementary material for: Phenolic compounds from pumpkin pulp: Extraction optimization and biological properties
Source: Food Chem X. 2024 Jul 6;23:101628. doi: 10.1016/j.fochx.2024.101628 (PMC11301232; doi:10.1016/j.fochx.2024.101628)
Supplement: Supplementary file 1 — Supplementary material [file mmc1.docx]

**Phenolic compounds from pumpkin pulp: extraction optimization and biological properties**

Nicola Pinna ^a,^^, Salwa Ben Abbou ^b,^^, Federica Ianni ^a,^, Giancarlo Angeles Flores ^c^, Anne Pietercelie ^b^, Giuseppe Italo Francesco Perretti ^d^, Francesca Blasi ^a,*^, Paola Angelini ^e^, Lina Cossignani ^a^

^a^ Department of Sciences and Techniques, Institut Meurice, 1070 Bruxelles, Belgium

^b^ Department of Pharmaceutical Sciences, University of Perugia, 06126 Perugia, Italy

^c^ Department of Chemistry, Biology and Biotechnology, University of Perugia, 06100 Perugia, Italy

^d^ Department of Agricultural, Food and Environmental Sciences, University of Perugia, 06121 Perugia, Italy

* Corresponding author: francesca.blasi@unipg.it; Tel.: +39-075-5857954.

^ These authors contributed equally to the manuscript


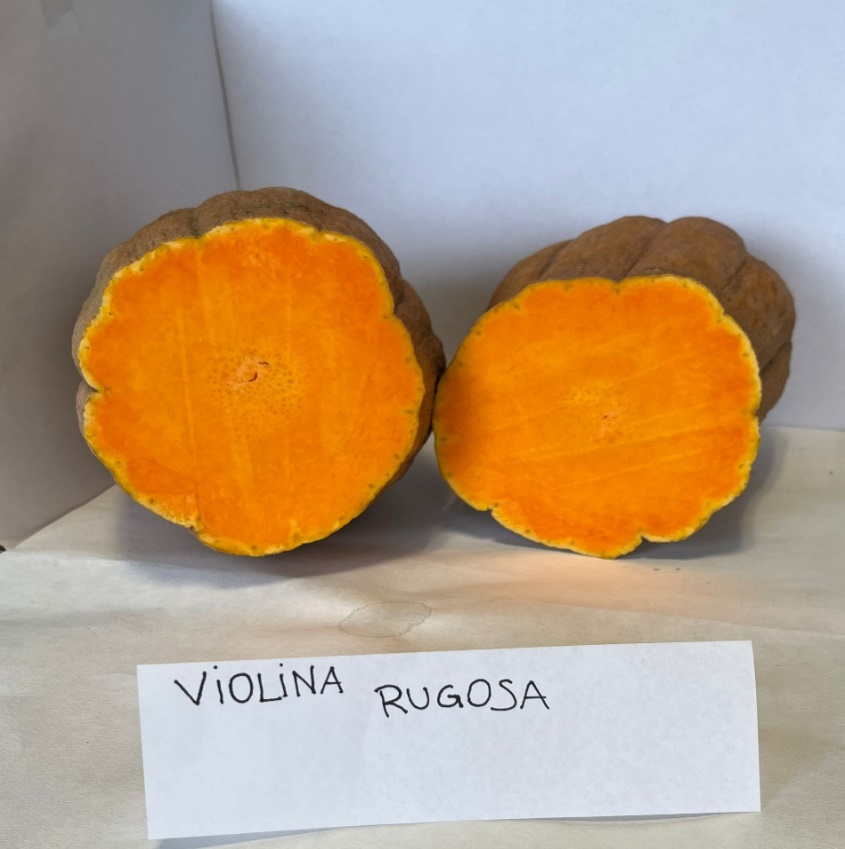


**Figure S1.** Images of pumpkins harvested in 2022


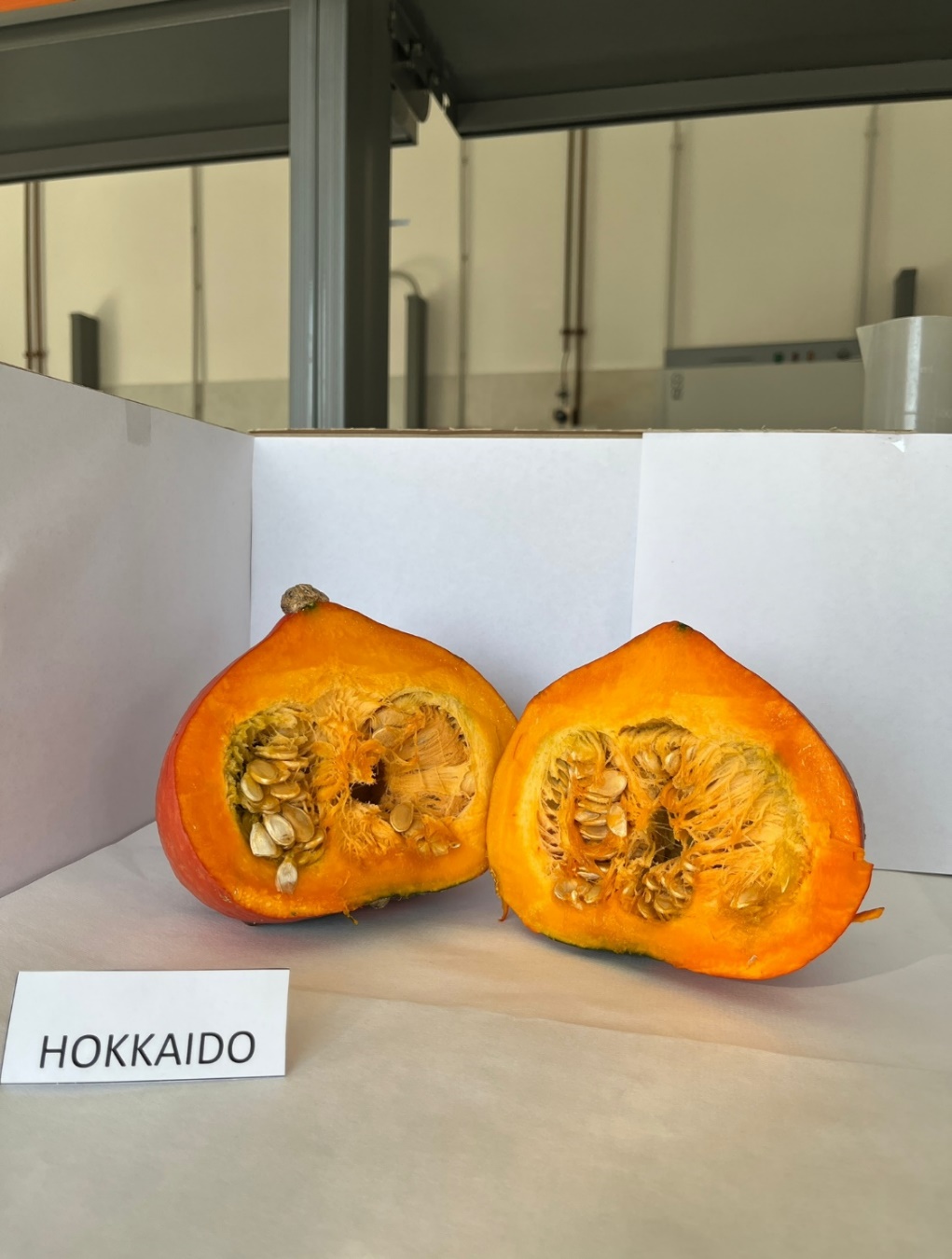

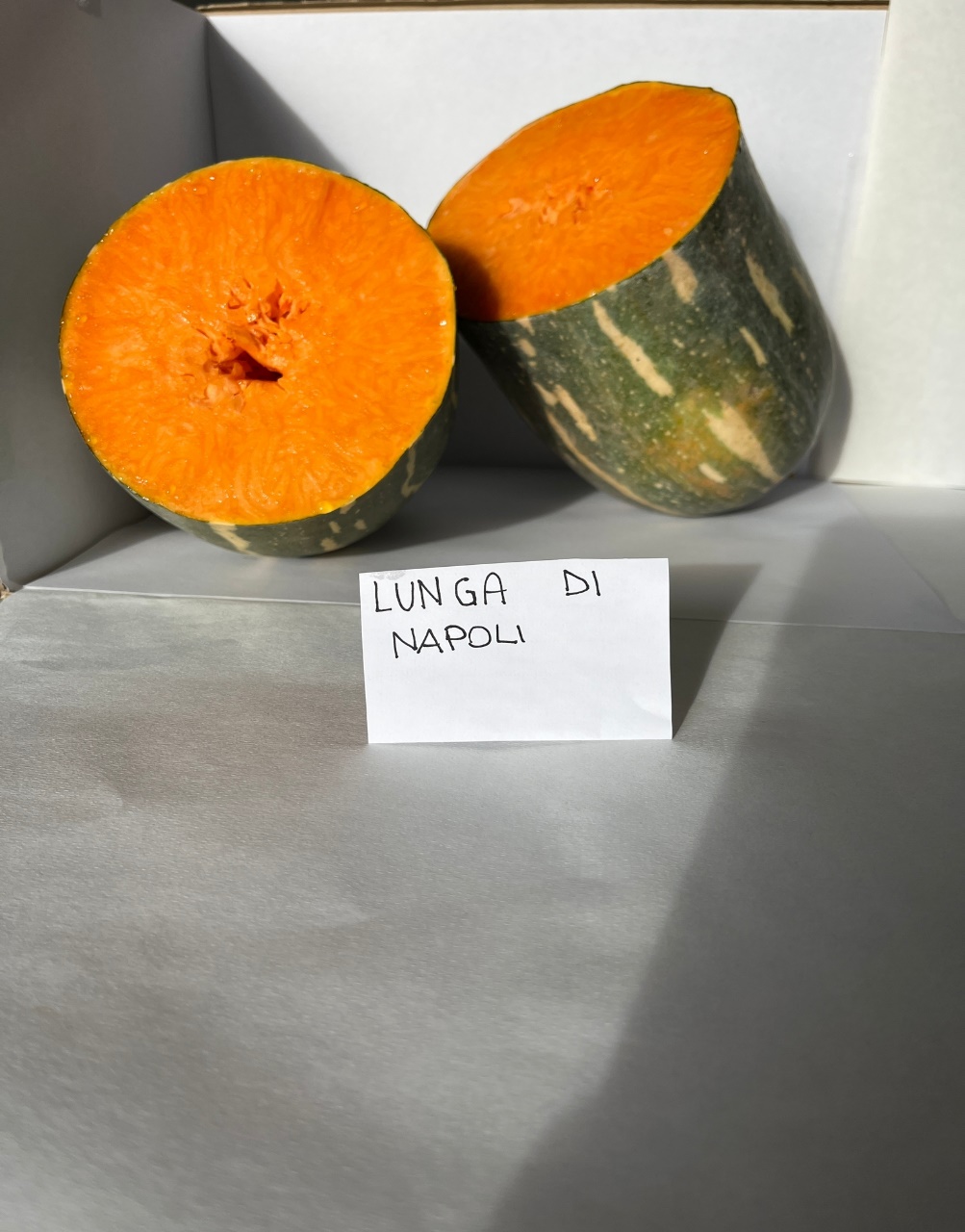

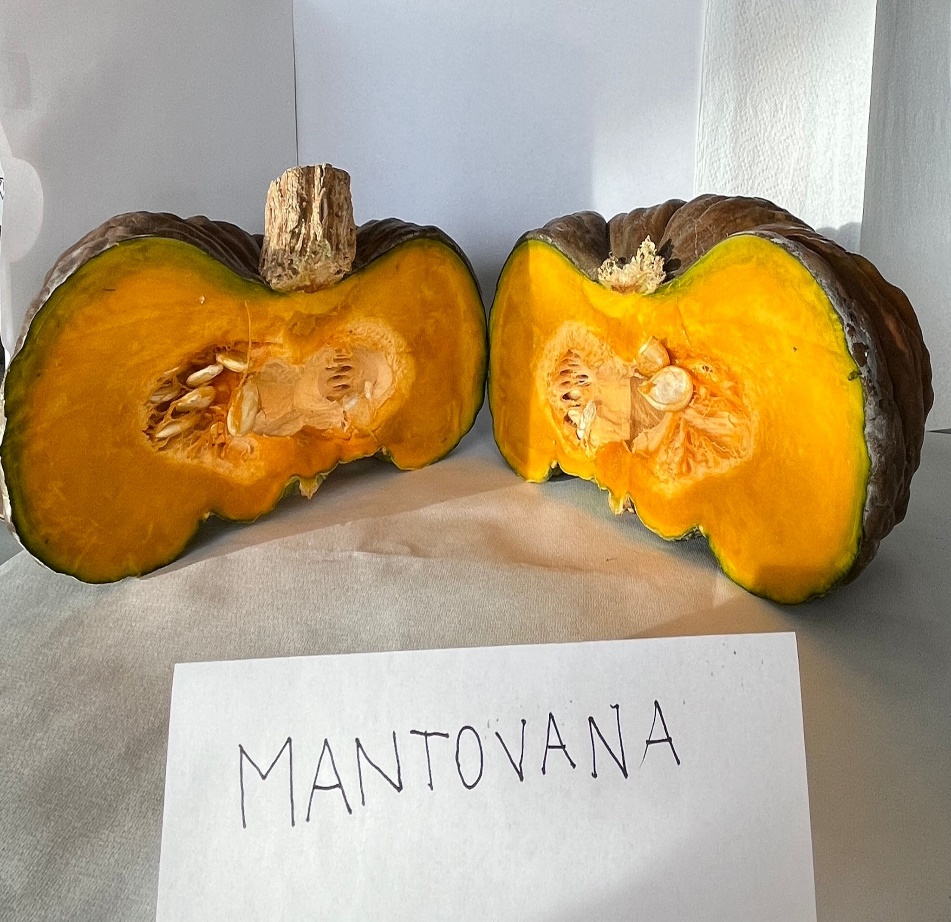

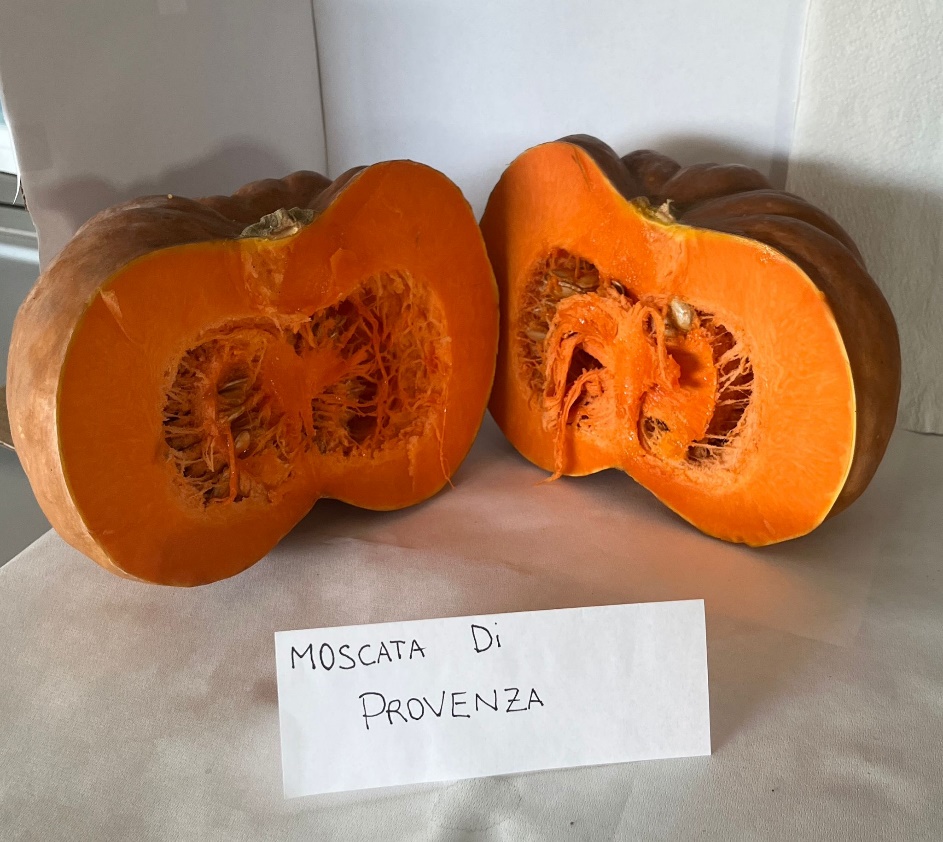


**Hokkaido**

**Lunga di Napoli**

**Mantovana**

**Moscata di Provenza**

**Violina rugosa**


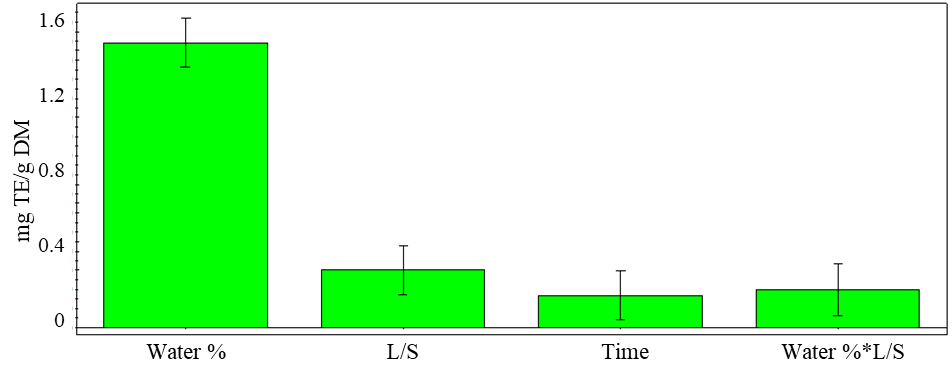

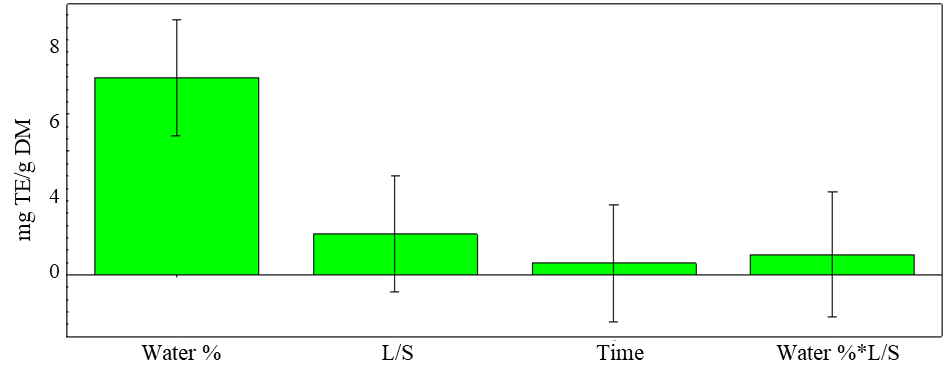


**d**

**c**


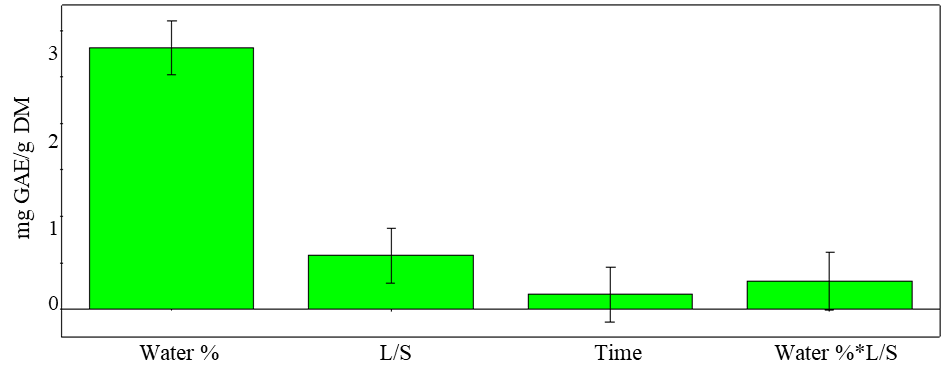


**a**

**b**

**
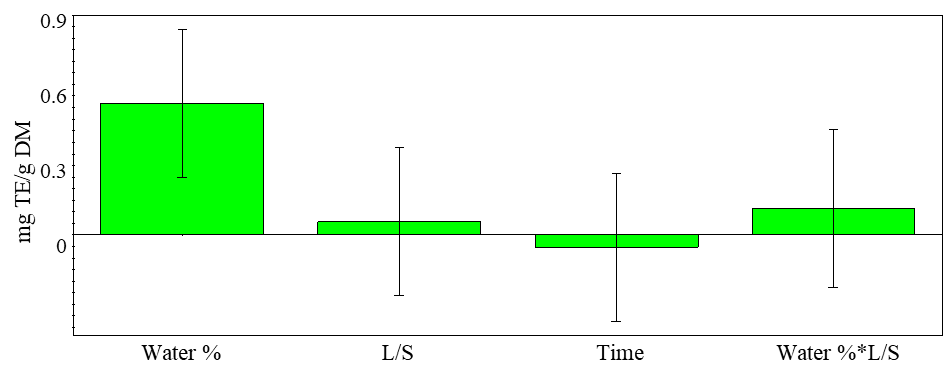
**

**Figure S2.** UAE experimental design. Coefficient plots showing the effect of water % in ethanol, liquid/solid ratio, time of extraction and interaction (% water * ratio) on the responses: a) TPC, b) DPPH, c) ABTS, and d) FRAP. L/S, liquid to solid ratio.


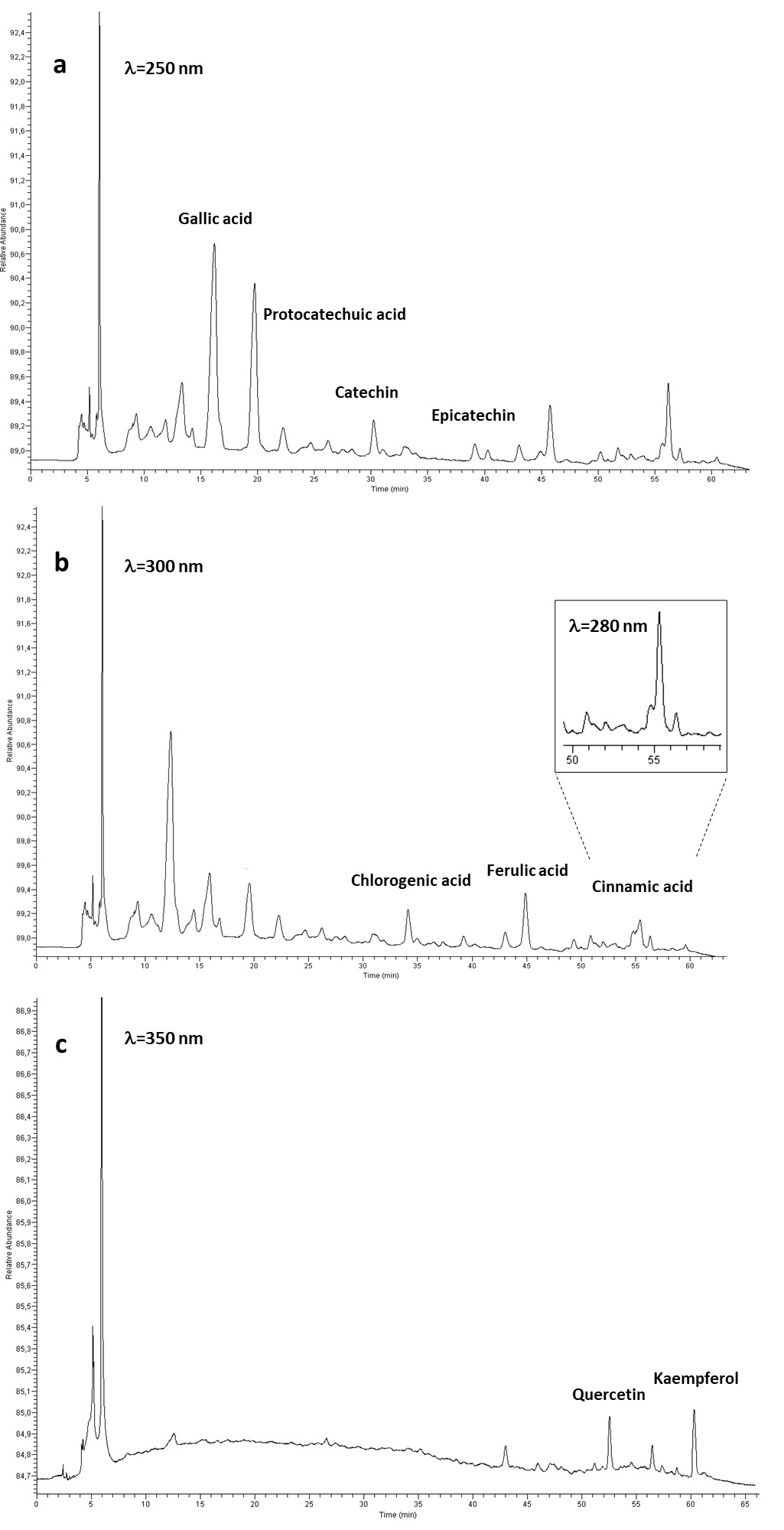


**Figure S3.** HPLC-DAD chromatograms of Moscata di Provenza extracted at 250 nm (A), 300 nm (B) and 350 nm (C).


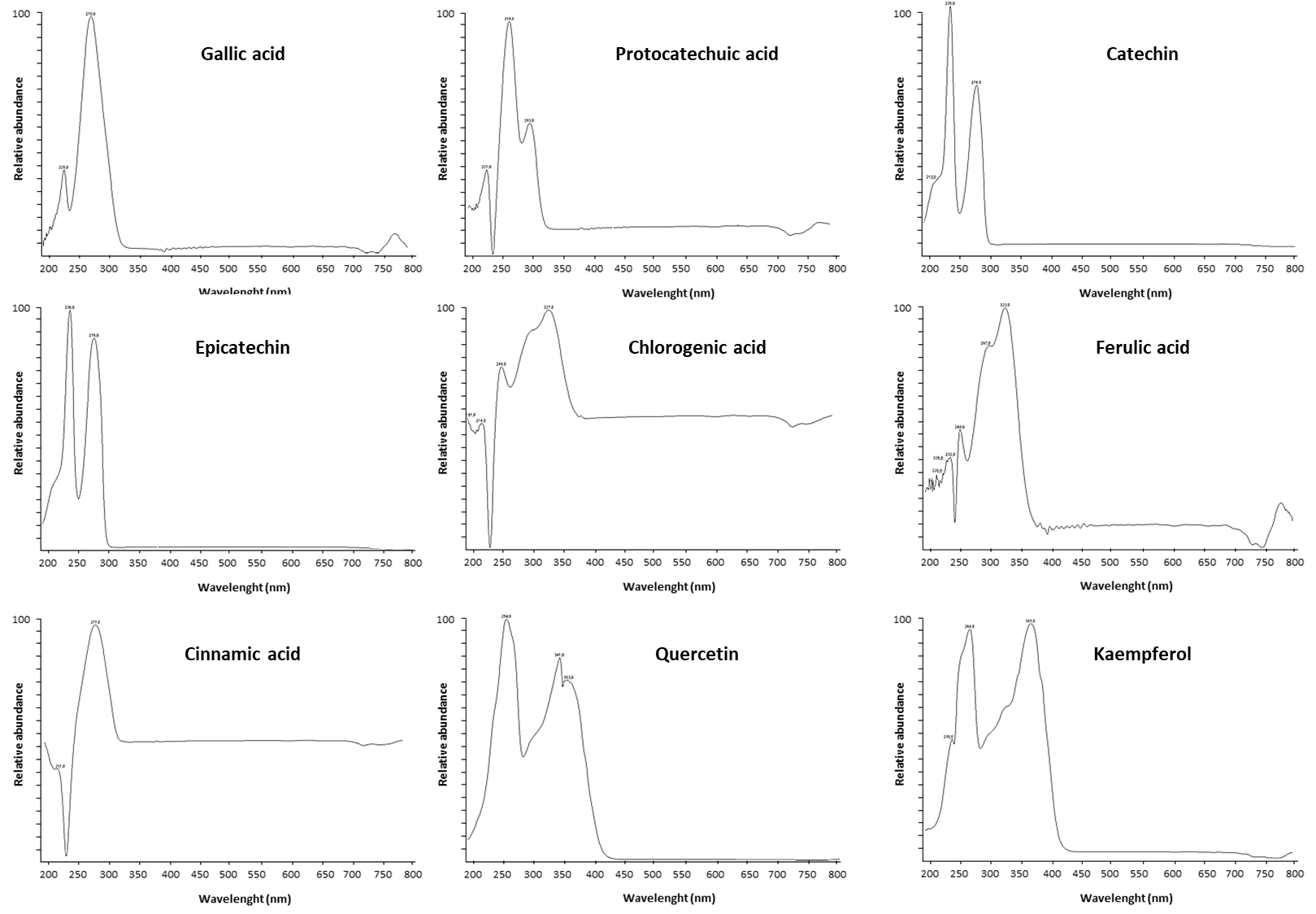


**Figure S4.** On-line DAD UV-Vis spectra of the identified compounds.

**2021**

**Figure S5.** Content of pulp phenols (μg/g), grouped as phenolic acids, flavonoids and hydroxycinnamic acids**,** of five pumpkin varieties harvested in 2021.

**2022**

**Figure S6.** Content of pulp phenols (μg/g), grouped as phenolic acids, flavonoids and hydroxycinnamic acids**,** of five pumpkin varieties harvested in 2022.

**Table S1.** Worksheet of the eleven experimental conditions (VRN1-VRN11) for UAE.

|  | **Water** | **L/S ratio** | **Time** |
| --- | --- | --- | --- |
|  | **(%)** | **(mL/g)** | **(min)** |
| VRN1 | 0 | 10 | 10 |
| VRN2 | 50 | 10 | 10 |
| VRN3 | 0 | 30 | 10 |
| VRN4 | 50 | 30 | 10 |
| VRN5 | 0 | 10 | 40 |
| VRN6 | 50 | 10 | 40 |
| VRN7 | 0 | 30 | 40 |
| VRN8 | 50 | 30 | 40 |
| VRN9 | 25 | 20 | 25 |
| VRN10 | 25 | 20 | 25 |
| VRN11 | 25 | 20 | 25 |
| Water (%), percentage of water in ethanol; L/S ratio, liquid/solid ratio; VRN, Violina rugosa number (where VR indicates the Violina rugosa variety and N the number of the experiment) | | | |

**Table S2.** Goodness of fit (R^2^) and goodness of predictability (Q^2^) coefficients of statistical models

|  | **R^2^** | **Q^2^** |
| --- | --- | --- |
| **TPC** | 0.997 | 0.830 |
| **DPPH** | 0.918 | 0.649 |
| **ABTS** | 0.976 | 0.738 |
| **FRAP** | 0.998 | 0.774 |
| **Gallic acid** | 0.849 | 0.501 |
| **Chlorogenic acid** | 0.863 | 0.599 |

**Table S3.** Model coefficients (Coe.), standard error (Std. Err.) and *p*-values for the considered responses.

|  | **Gallic acid** | | | | **Chlorogenic acid** | | | |
| --- | --- | --- | --- | --- | --- | --- | --- | --- |
|  | Coef. | Std. Err. | *p*-value | Coef. | | Std. Err. | *p*-value |  |
| Constant | 191.565 | 29.2663 | 0.007249 | 145.405 | | 13.0165 | 0.001537 |  |
| Water % | 118.563 | 31.2869 | 0.032233 | 59.168 | | 13.9152 | 0.023840 |  |
| Ratio | 18.0217 | 31.2869 | 0.604977 | 11.7313 | | 13.9152 | 0.461106 |  |
| Time | -43.6143 | 31.2869 | 0.257624 | -0.8621 | | 13.9152 | 0.954493 |  |
| Wat %*L/S | 17.7523 | 33.4471 | 0.632368 | 5.89842 | | 14.876 | 0.718252 |  |

**Table S4.** Correlation analysis (coefficient of determination, R^2^) for the eleven samples (VNR1-VNR-11) between the spectrophotometric parameters (TPC, DPPH, ABTS, FRAP) and TPC determined by HPLC.

|  | **TPC** | **DPPH** | **ABTS** | **FRAP** | **TPC-HPLC** |
| --- | --- | --- | --- | --- | --- |
| **TPC** | - |  |  |  |  |
| **DPPH** | 0.8245 | - |  |  |  |
| **ABTS** | 0.9808 | 0.7652 | - |  |  |
| **FRAP** | 0.9878 | 0.9575 | 0.8299 | - |  |
| **TPC-HPLC** | 0.8465 | 0.8343 | 0.8176 | 0.7853 | - |

**Table S5.** Chromatographic and spectral features of phenolic compounds

| **Compound** | **Rt (min)** | **UV-Vis spectrum (λ_max_, nm)** |
| --- | --- | --- |
| Gallic acid | 15.62 | 213, 235, 270 |
| Protocatecuic acid | 21.43 | 221, 259, 293 |
| Catechin | 30.82 | 213, 235, 270 |
| Chlorogenic acid | 34.47 | 248, 315, 327 |
| Epicatechin | 38.43 | 213, 236, 276 |
| Ferulic acid | 45.87 | 248, 297, 330 |
| Quercetin | 51.22 | 254, 341, 353 |
| Cinnamic acid | 56.46 | 211, 278 |
| Kaempferol | 60.08 | 235, 264, 365 |

**Table S6.** Correlation analysis (coefficient of determination, R^2^) for pumpkin pulp extracts between the spectrophotometric parameters (TPC, DPPH, ABTS, FRAP) and TPC determined by HPLC (TPC-HPLC).

|  | **TPC** | **DPPH** | **ABTS** | **FRAP** | **TPC-HPLC** |
| --- | --- | --- | --- | --- | --- |
| **TPC** | - |  |  |  |  |
| **DPPH** | 0.6906 | - |  |  |  |
| **ABTS** | 0.7734 | 0.3459 | - |  |  |
| **FRAP** | 0.7918 | 0.9307 | 0.4962 | - |  |
| **TPC-HPLC** | 0.9010 | 0.6378 | 0.5752 | 0.7282 | - |

**Table S7.** PCA: eigenvalue, percentage of variance and cumulative percentage

|  | **Eigenvalue** | **Variability (%)** | **Cumulative %** |
| --- | --- | --- | --- |
| F1 | 4.950 | 82.507 | 82.507 |
| F2 | 0.576 | 9.604 | 92.110 |
| F3 | 0.322 | 5.362 | 97.472 |
| F4 | 0.127 | 2.111 | 99.583 |
| F5 | 0.017 | 0.288 | 99.872 |
| F6 | 0.008 | 0.128 | 100 |

**Table S8.** LDA: prior and posterior classification, membership probabilities, scores and squared distances

| **Observation** | **Prior** | **Posterior** | **Pr(2021)** | **Pr(2022)** | **F1** | **D²(2021)** | **D²(2022)** |
| --- | --- | --- | --- | --- | --- | --- | --- |
| Obs1 | 2021 | 2022 | 1.000 | 0.000 | -6.035 | 7.532 | 147.183 |
| Obs2 | 2021 | 2022 | 1.000 | 0.000 | -5.217 | 5.291 | 126.018 |
| Obs3 | 2021 | 2022 | 1.000 | 0.000 | -7.527 | 6.555 | 180.727 |
| Obs4 | 2021 | 2022 | 1.000 | 0.000 | -4.363 | 6.015 | 106.971 |
| Obs5 | 2021 | 2022 | 1.000 | 0.000 | -5.784 | 7.621 | 141.462 |
| Obs6 | 2021 | 2022 | 0.000 | 1.000 | 4.430 | 110.186 | 7.672 |
| Obs7 | 2021 | 2022 | 0.000 | 1.000 | 5.807 | 138.172 | 3.804 |
| Obs8 | 2021 | 2022 | 0.000 | 1.000 | 5.987 | 146.128 | 7.576 |
| Obs9 | 2021 | 2022 | 0.000 | 1.000 | 6.207 | 146.854 | 3.228 |
| Obs10 | 2021 | 2022 | 0.000 | 1.000 | 6.495 | 156.856 | 6.568 |

**Table S9.** LDA: matrix for the training sample (above) and for the cross-validation results (below)

| Classification results: training sample | | | | |
| --- | --- | --- | --- | --- |
| from\to | 2021 | 2022 | Total | % correct |
| 2021 | 5 | 0 | 5 | 100.00% |
| 2022 | 0 | 5 | 5 | 100.00% |
| Total | 5 | 5 | 10 | 100.00% |
|  |  |  |  |  |
| Classification results: cross-validation | | | | |
| from\to | 2021 | 2022 | Total | % correct |
| 2021 | 4 | 1 | 5 | 80.00% |
| 2022 | 0 | 5 | 5 | 100.00% |
| Total | 4 | 6 | 10 | 90.00% |
